# The Flexible MWCNTs-PDMS Electrode-Based Counter-Rotating Hybrid Triboelectric–Electromagnetic Nanogenerator for Effective Wind Energy Harvesting

**DOI:** 10.3390/polym18212754

**Published:** 2026-11-05

**Authors:** Yao Zhu, Xue Liu, Wei Zhong, Jiwen Fang, Chong Li, Xiaohong Dong, Jiang Shao

**Affiliations:** 1 The College of Mechanical Engineering, Jiangsu University of Science and Technology, Zhenjiang 212000, Chinafjw617@just.edu.cn (J.F.); lichong@just.edu.cn (C.L.); 2 The College of Chemistry and Molecular Sciences, Henan University, Kaifeng 475001, China; xliu@henu.edu.cn; 3 Yangzhou Chenhua New Materials Co., Ltd., Yangzhou 225000, China

**Keywords:** triboelectric nanogenerators, electro-magnetic generator, MWCNTs, PDMS, polymer, wind energy collection

## Abstract

Wind energy is one of the most abundant and sustainable renewable energy resources. However, its efficient utilization remains challenging because of fluctuating wind speeds and multidirectional airflow. In this work, we develop a dual-rotor hybrid triboelectric–electromagnetic energy harvester (H-TENG) featuring flexible multiwalled carbon nanotube/poly(dimethylsiloxane) (MWCNTs/PDMS) composite electrodes to improve wind energy harvesting efficiency. The effects of electrode materials and rotor configurations on the electrical output of the device are systematically investigated. Compared with conventional copper electrodes, the flexible MWCNTs/PDMS composite electrodes increase the peak output power of the triboelectric unit by 46.1% at a wind speed of 6 m s^−1^, while the dual-rotor configuration further enhances the peak output power by up to approximately 195.6% relative to the single-rotor design. To overcome the limited, short-circuit current output of the triboelectric generator, an electromagnetic generator is integrated into the hybrid system. The optimized device delivers a maximum output open-circuit voltage of 1212.34 V from the TENG and a maximum short-circuit current of 13.64 mA from the EMG. The harvested energy is sufficient to continuously power a digital watch and 240 light-emitting diodes (LEDs). This work provides an effective material–structure synergistic strategy for high-performance hybrid wind energy harvesters and offers a promising approach for harvesting low-speed and multidirectional wind energy.

## 1. Introduction

The extensive utilization of fossil fuels has caused serious environmental concerns, accelerating the development of renewable and clean energy harvesting technologies [1,2,3,4]. Among various natural energy resources, wind energy has attracted considerable attention owing to its sustainability, wide distribution, and abundant reserves [5,6,7,8]. Various wind energy harvesting technologies, including electromagnetic generators (EMGs), piezoelectric generators, triboelectric nanogenerators (TENGs), and hybrid energy harvesters, have been developed to convert wind energy into electricity [9,10,11,12,13,14,15,16]. However, conventional electromagnetic and piezoelectric wind energy harvesters generally suffer from bulky structures, high fabrication costs, and poor energy conversion efficiency under low-speed wind conditions [17,18,19,20,21,22,23]. Therefore, developing an efficient, lightweight, and low-cost wind energy harvesting technology remains highly desirable.

As an emerging mechanical energy conversion technology, TENGs have attracted considerable interest because of their lightweight design, simple fabrication, low cost, and excellent adaptability to low-frequency mechanical motions [24,25,26,27,28,29,30,31,32,33]. Consequently, TENGs have been widely investigated for harvesting various forms of ambient mechanical energy, including wind, ocean waves, and human motion. For wind-driven TENGs, the electrical output is strongly influenced by the effective contact between triboelectric layers, charge transport capability, and structural durability during continuous operation [34,35,36,37]. Compared with conventional rigid metal electrodes, flexible polymer-based conductive composites can effectively improve conformal contact, enlarge the effective contact area, and maintain stable electrical pathways under repeated deformation. For example, Shin et al. [36] developed a porous carbon nanotube/silicone rubber composite sponge-based triboelectric nanogenerator, in which conductive carbon nanotube networks facilitated charge transport while the porous elastomer structure increased the effective contact area, achieving an output open-circuit voltage of 1120 V and a short-circuit current of 25.6 μA under a driving force of 7 N. Despite these encouraging advances, the application of flexible-polymer composite electrodes in rotary wind-driven TENGs remains limited, and the synergistic optimization of electrode flexibility, structural robustness, and electrical output has rarely been systematically explored.

Existing wind-driven TENGs can generally be divided into vibration-driven and rotation-driven configurations [38,39,40,41,42]. Vibration-driven devices convert wind energy into electricity through wind-induced oscillation, which periodically drives contact–separation between triboelectric layers [40]. For example, Huang et al. [38] reported a wind-bell-inspired triboelectric nanogenerator (W-TENG) for extremely low-speed and omnidirectional wind energy harvesting. The experimental results indicated that the W-TENG could generate electrical output at a wind speed as low as 0.5 m s^−1^ and realize omnidirectional wind energy harvesting over the range of 0–360°. After parameter optimization, the W-TENG achieved a maximum output open-circuit voltage of 9.3 V and a maximum output short-circuit current of 0.63 μA. In contrast, rotation-driven TENGs harvest wind energy through continuous rotational contact or sliding motion, generally providing higher output stability and energy conversion efficiency. Nevertheless, the peak output power and short-circuit current of existing rotary TENGs remain insufficient for directly powering practical electronic devices, particularly under low-speed wind conditions. Furthermore, most reported studies have focused primarily on either material optimization or structural improvement, while the synergistic integration of flexible conductive electrodes, high-efficiency rotating architectures, and short-circuit current-enhancement strategies has received relatively little attention. Therefore, it remains highly desirable to develop a high-performance hybrid wind energy harvesting system through simultaneous optimization of material selection and structural design.

In this work, we develop a hybrid triboelectric–electromagnetic wind energy harvester (H-TENG) by integrating flexible MWCNTs/PDMS composite electrodes with an inner–outer counter-rotating architecture for efficient wind energy harvesting. The proposed system consists of a TENG and an EMG. Flexible MWCNTs/PDMS composite electrodes are employed to replace conventional metal electrodes, where interconnected MWCNT conductive networks provide efficient electron transport, while the elastomeric PDMS matrix improves electrode flexibility, interfacial conformity, and structural durability during continuous sliding. Meanwhile, the counter-rotating inner and outer rotors significantly increase the relative sliding velocity between the triboelectric layers, thereby enhancing charge generation and electrical output. To compensate for the relatively low short-circuit current characteristic of TENG, an EMG is further integrated into the system to improve the overall power supply capability. The influences of electrode materials and rotor configurations on the electrical output performance are systematically investigated. The optimized H-TENG achieves a maximum TENG open-circuit voltage of 1212.34 V, while the EMG short-circuit current reaches a maximum of 13.64 mA. The harvested energy is sufficient to continuously power a digital watch and 240 commercial light-emitting diodes (LEDs). This work provides a material–structure synergistic strategy for improving the performance of hybrid triboelectric–electromagnetic wind energy harvesters and offers a promising approach toward efficient low-speed wind energy harvesting and self-powered distributed sensing systems.

## 2. Materials and Methods

### 2.1. Materials

The PDMS (SYLGARD™ 184 Silicone Elastomer Kit) used in this study was purchased from Dow Corning (Midland, MI, USA). MWCNTs (outer diameter: 8–15 nm; length: ~50 μm) were supplied by Shenzhen Nano Technology Co., Ltd. (Shenzhen, China). Xylene was purchased from Shanghai Macklin Biochemical Co., Ltd. (Shanghai, China). All chemicals were used as received without further treatment.

### 2.2. Fabrication of H-TENG

The framework of the H-TENG was fabricated using a Bambu Lab A1 3D printer with polylactic acid (PLA) as the printing material (Shenzhen Bambu Lab Technology Co., Ltd., Shenzhen, China). The outer rotor was designed as a hollow cylindrical structure with a diameter of 200 mm and a wall thickness of 3 mm. Two copper rings were attached to the upper and lower ends of the outer rotor to serve as current-collecting electrodes for electrical output. A 0.25 mm thick polyethylene terephthalate (PET) film was bonded to its inner surface, followed by the uniform arrangement of twelve flexible composite electrodes (48 mm × 66 mm × 0.25 mm) with a spacing of 4 mm between adjacent electrodes. A 0.05 mm thick polytetrafluoroethylene (PTFE) film was laminated onto each electrode to serve as the triboelectric layer, while wind-harvesting blades were mounted on the outer surface of the rotor. The inner rotor was fabricated as a conical structure with a cone angle of 90° and a height of 60 mm, in which 0.2 mm thick polyamide (PA) films with a length of 125 mm were inserted into the vertical grooves. The assembled inner rotors were mounted on straight shafts equipped with stoppers to prevent axial displacement. The straight shafts were connected to a central sleeve (70 mm in diameter and 2.5 mm in wall thickness), inside which six permanent magnets were embedded, while the upper wind-harvesting assembly was fixed to the sleeve. Finally, the outer rotor and inner rotors were assembled onto the central shaft, and six copper coils (495 turns, 19 mm × 12 mm) were positioned around the magnets to construct the electromagnetic generation unit, completing the fabrication of the H-TENG.

### 2.3. Fabrication of MWCNTs/PDMS Nanocomposite

The MWCNTs/PDMS flexible conductive film was prepared through a solution-assisted dispersion and blade-coating process. Briefly, 0.9 g of MWCNTs was dispersed in 30 mL of xylene using an ultrasonic cell disruptor (Scientz-IID, Ningbo Scientz Biotechnology Co., Ltd., Ningbo, China) for 5 min. Meanwhile, 15 g of Sylgard 184 PDMS base agent (Part A) was mixed with 30 mL of xylene and ultrasonically dispersed under the same conditions. The two suspensions were subsequently combined, and the beakers were rinsed with xylene to ensure complete transfer of the MWCNTs. The resulting mixture was further ultrasonically treated for 6 min to obtain a homogeneous precursor, followed by heating at 65 °C in a well-ventilated environment until the xylene was completely evaporated and the remaining mass reached approximately 15.9 g. After cooling to room temperature, 1.5 g of Sylgard 184 curing agent (Part B) was added, and the mixture was stirred for 1 min to obtain the coating precursor. A PTFE film was then fixed onto a glass substrate and uniformly coated with the MWCNTs/PDMS precursor using a film applicator. The coating thickness was controlled at 250 μm, which had been identified as the optimum value in preliminary experiments [43]. Finally, the coated sample was cured at 55 °C for 6 h in an oven to obtain a compact and flexible MWCNTs/PDMS conductive film. Following the same procedure, flexible electrodes containing different MWCNT loadings of 4, 6, 8, and 10 wt% were prepared by adjusting the amount of MWCNTs while keeping the other preparation conditions consistent. The MWCNT content was defined as the weight percentage of MWCNTs relative to the total mass of MWCNTs, PDMS base, and curing agent.

### 2.4. Characterization of MWCNTs/PDMS Nanocomposite

The morphology of the MWCNTs/PDMS nanocomposite was characterized using field-emission scanning electron microscope (Carl Zeiss, Jena, Germany).

### 2.5. Electrical Measurement

A commercial anemometer (GM618) was employed to continuously monitor the wind speed, ensuring stable experimental conditions throughout the measurements. The electrical output signals were acquired and recorded using a digital multimeter (Agilent 34401A, Agilent Technologies, Santa Clara, CA, USA).

## 3. Results and Discussion

**Figure 1:**
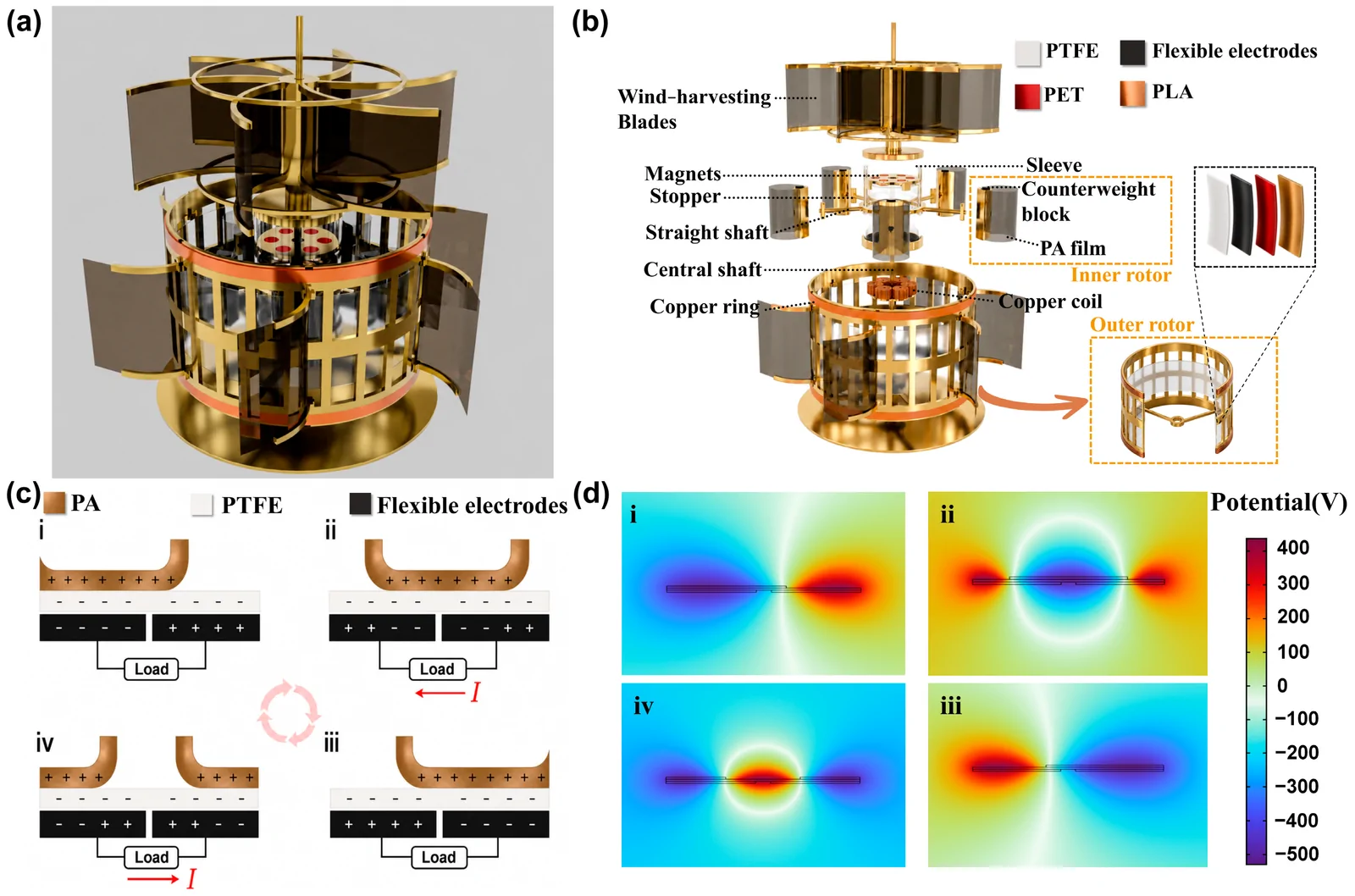
Structure and illustration of the working principle of the H-TENG. (**a**) Schematic diagram of the H-TENG. (**b**) Enlarged view of the H-TENG. (**c**) Schematic illustration of the working principle of the TENG. (**d**) The corresponding potential distribution calculated by COMSOL in a two-dimensional plane.

The overall configuration of the proposed H-TENG is illustrated in Figure 1a,b. The H-TENG integrates a TENG and an EMG, and consists of an outer rotor together with five symmetrically distributed inner rotors. To improve the electrical output and mechanical durability of the TENG, flexible MWCNTs/PDMS composite electrodes are employed to replace conventional rigid copper electrodes. Flexible MWCNTs/PDMS composite electrodes were fabricated by solution blending followed by film coating and thermal curing, as illustrated in Figure S1 (Supporting Information). The interconnected MWCNT conductive network provides efficient electron transport, while the elastomeric PDMS matrix endows the electrode with excellent flexibility and structural stability during repeated sliding deformation. In addition, Figure S2 (Supporting Information) shows that PTFE and PA are located at opposite ends of the triboelectric series, indicating a large triboelectric polarity difference. Therefore, PTFE and PA were selected as the triboelectric pair to enhance charge generation during the sliding process. As shown in Figure 1a,b, the outer rotor consists of a cylindrical PLA framework covered with PET film, flexible MWCNTs/PDMS composite electrodes and PTFE films, while each inner rotor is composed of a conical PLA skeleton, a PA film, and a counterweight block. The five inner rotors are supported by a straight shaft equipped with mechanical stoppers to prevent excessive radial displacement. Owing to the flexibility of the PA films, the contact area between the PA and PTFE layers can automatically increase with increasing wind speed as the centrifugal force drives the inner rotors to expand outward. Meanwhile, the upper and lower wind-harvesting assemblies rotate in opposite directions, causing the inner and outer rotors to rotate counter to each other. This counter-rotating configuration significantly increases the relative sliding velocity between the triboelectric layers, thereby improving the electrical output of the TENG. In addition, the EMG consists of six permanent magnets mounted on the rotating sleeve and six stationary copper coils fixed around the central shaft. The relative motion between the magnets and coils generates electromagnetic induction to compensate for the relatively low current output of the triboelectric unit.

The electricity generation mechanism of the TENG is schematically illustrated in Figure 1c. When the device is driven by wind, continuous sliding occurs between the PTFE and PA triboelectric layers. Owing to their large electronegativity difference, electrons transfer from the PA film to the PTFE film, leaving the PTFE surface negatively charged and the PA surface positively charged (Figure 1(ci)). As the PA film slides from electrode A toward electrode B, the electrostatic equilibrium is disturbed, driving electrons to flow through the external circuit from electrode A to electrode B and generating an electrical current (Figure 1(cii)). When the PA film completely overlaps electrode B, the induced charge on electrode B reaches its maximum value (Figure 1(ciii)). As the sliding motion continues, electrons flow back in the opposite direction (Figure 1(civ)), producing a periodic alternating-current (AC) output under continuous wind excitation.

To further verify the working mechanism, the electric potential distribution of the TENG was simulated using the finite-element method in COMSOL Multiphysics version 6.3, and the corresponding results are presented in Figure 1d. The simulated potential contours clearly reveal the evolution of the potential difference between the two electrodes during the sliding process, which agrees well with the proposed triboelectrification and electrostatic induction mechanism. The COMSOL simulation is employed to qualitatively visualize the variation in electric potential distribution during the contact–separation process and to verify the charge-transfer mechanism, rather than to quantitatively predict the experimental output open-circuit voltage [44,45]. Therefore, differences between the simulated and experimental values may arise from the simplified geometry and idealized boundary conditions, as well as the absence of dynamic deformation, variable effective contact area, and charge accumulation in the electrostatic model.

**Figure 2:**
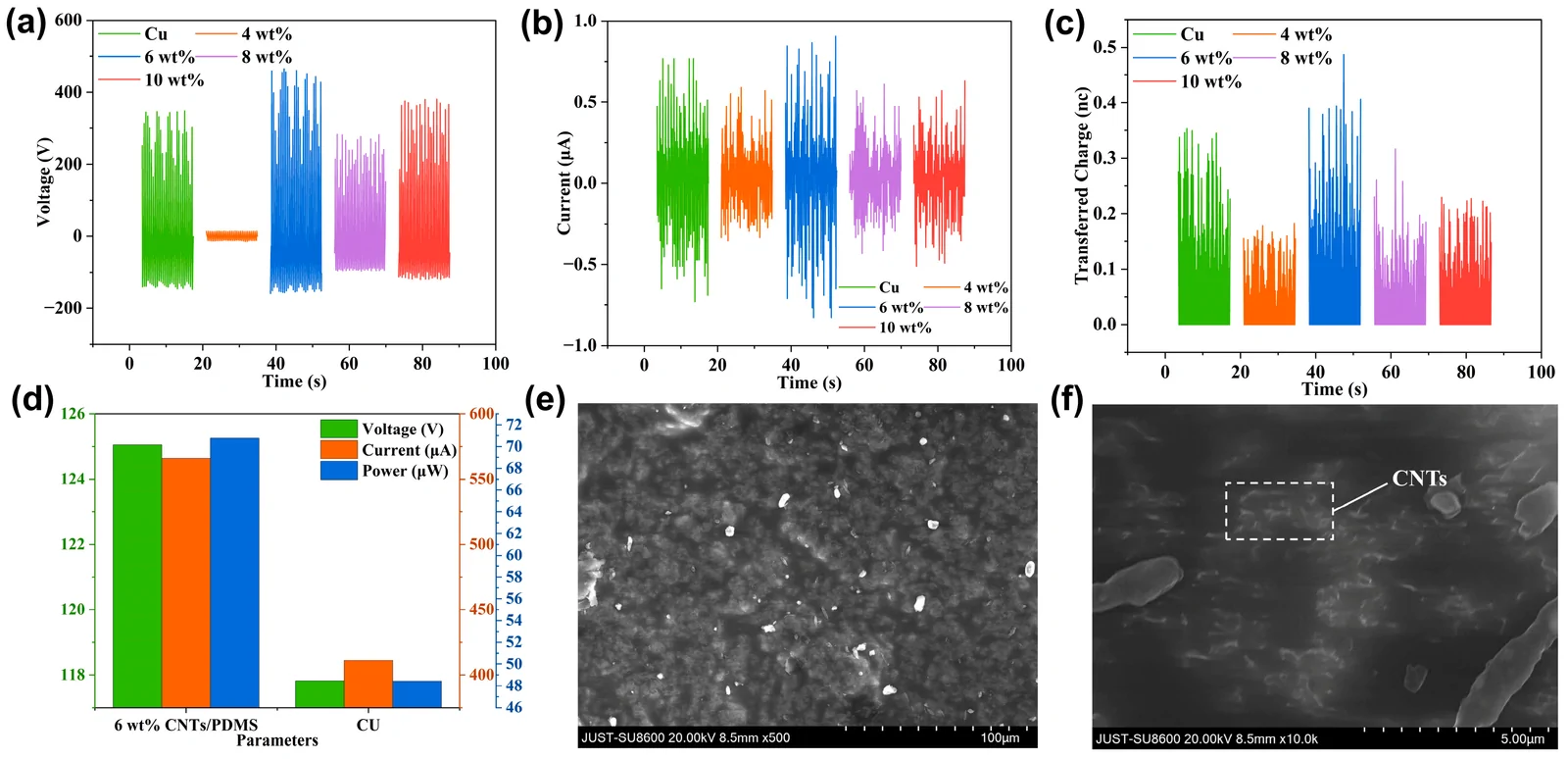
The output performance of different electrode materials of the TENG. (**a**) The open-circuit voltage, (**b**) short-circuit current, and (**c**) transferred charges with different electrode materials of the TENG. (**d**) Comparison of output between the 6 wt% MWCNTs/PDMS flexible electrode and the copper electrode. (**e**) SEM surface scan of a flexible electrode. (**f**) SEM surface scan of a flexible electrode coated with PDMS dielectric layer.

Previous study has demonstrated that electrode materials and their interfacial electrical characteristics play a crucial role in determining the output performance of TENGs [46]. To investigate the influence of electrode materials on the electrical performance of the TENG, the outer rotor is fixed while the inner rotor is driven by a controlled airflow generated using electric fans. The wind speed is continuously monitored using a commercial anemometer and is maintained at 5 m s^−1^ throughout the measurements. The electrical output is evaluated using an inner rotor equipped with a PA triboelectric layer (125 mm × 55 mm × 0.05 mm). As shown in Figure 2a–c, the electrical output of the TENG strongly depends on the MWCNT content in the flexible composite electrode. To further understand this trend, the electrical resistance and conductivity of the MWCNTs/PDMS electrodes are characterized. As shown in Figure S3, as the MWCNT content increases from 4 to 10 wt%, the resistance decreases from 421.48 to 24.39 kΩ, while the conductivity increases from 0.0285 to 0.492 S m^−1^. However, the triboelectric output peaks at 6 wt% rather than at the highest conductivity. This behavior may be attributed to the increased stiffness at higher MWCNT loadings, as reported by Sidhu et al. [47], which potentially reduces electrode conformability and interfacial contact. Thus, the 6 wt% MWCNTs/PDMS electrode provides a favorable balance between electrical conductivity and mechanical flexibility. The optimized flexible electrode delivers a maximum open-circuit voltage of 465.02 V, together with the highest short-circuit current and transferred charge.

To further evaluate the advantage of the optimized flexible electrode, the electrical performance of the 6 wt% MWCNTs/PDMS composite electrode is compared with that of a conventional copper electrode under identical experimental conditions, as shown in Figure 2d–f. The experimental results (Figure 2d) show that the flexible composite electrode consistently exhibits superior electrical output, and the peak output power of the TENG increases by approximately 46.1% compared with that obtained using the copper electrode. Although copper possesses excellent intrinsic electrical conductivity, its high rigidity limits conformal contact with the triboelectric layer during repeated sliding, thereby reducing the effective contact area and charge collection efficiency. In contrast, the elastomeric PDMS matrix enables intimate interfacial contact throughout continuous deformation, while the embedded MWCNT conductive network provides efficient electron transport pathways. The corresponding SEM images (Figure 2e,f) further support this mechanism. Specifically, the low-magnification image (Figure 2e) reveals a relatively smooth surface with uniformly distributed MWCNTs that increase the effective contact area during sliding, whereas the high-magnification image (Figure 2f) clearly demonstrates the formation of a continuous interconnected MWCNT conductive network within the PDMS matrix. The synergistic combination of enhanced interfacial conformity and efficient electron transport therefore improves charge collection efficiency, leading to the superior electrical output of the optimized 6 wt% MWCNTs/PDMS composite electrode. SEM images acquired before and after electricity generation (Figure S6) show no obvious morphological changes under the tested operating conditions, indicating that the surface morphology of the MWCNTs/PDMS composite electrode remains largely unchanged during operation.

To maximize the electrical output of the TENG, the influences of the triboelectric layer configuration and rotor architecture are systematically investigated. Specifically, the effects of the PA film thickness, triboelectric contact area, and the number of inner rotors are evaluated under identical operating conditions to identify the optimal structural parameters and elucidate their underlying mechanisms.

**Figure 3:**
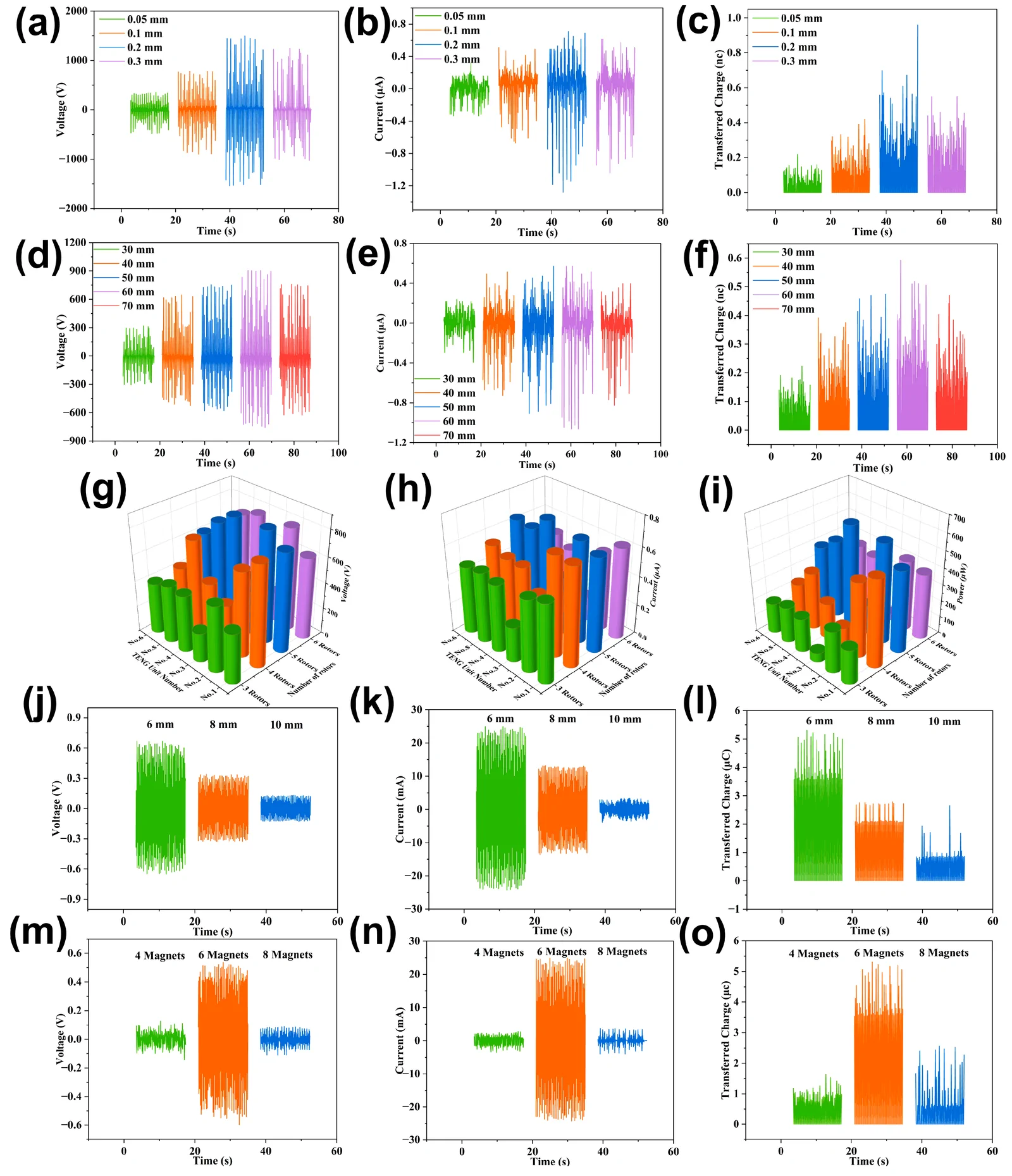
The impact of structural parameters on the electrical performance of the H-TENG. (**a**) Open-circuit voltage, (**b**) short-circuit current, and (**c**) transferred charges of single TENG unit with different blade thicknesses. (**d**) Open-circuit voltage, (**e**) short-circuit current, and (**f**) transferred charges of single TENG unit with different widths. (**g**) Open-circuit voltage, (**h**) short-circuit current, and (**i**) transferred charges of TENG unit with different inner rotors. (**j**) Open-circuit voltage, (**k**) short-circuit current, and (**l**) transferred charges of EMG with different distances between the magnets and the coils. (**m**) Open-circuit voltage, (**n**) short-circuit current, and (**o**) transferred charges of EMG with different distances between the magnets and the coils.

The thickness of the PA film is first optimized while maintaining the wind speed and PA film width at 5 m s^−1^ and 50 mm, respectively. PA films with thicknesses of 0.05, 0.1, 0.2, and 0.3 mm are evaluated under identical experimental conditions. As shown in Figure 3a–c, the open-circuit voltage, short-circuit current, and transferred charge initially increase and then decrease as the PA film thickness increases. The maximum electrical output is achieved at a PA thickness of 0.2 mm, corresponding to an open-circuit voltage of 1495.49 V, a short-circuit current of 0.71 μA, and a transferred charge of 0.96 nC. A previous study has shown that triboelectric-layer thickness affects interfacial deformation and effective contact area [48]. A thinner PA film provides insufficient structural stability for stable contact with the PTFE surface, whereas excessive thickness reduces its deformability and effective interfacial contact. Therefore, a PA thickness of 0.2 mm provides an appropriate balance between flexibility, structural stability, and effective contact, resulting in the highest electrical output. Previous studies have indicated that the area of triboelectric layers has a significant influence on the output performance of TENG [49,50]. To reveal its influence in the present device, the width of the PA film is varied while its length and thickness are maintained at 125 mm and 0.2 mm, respectively. The measurements are performed under a constant wind speed of 5 m s^−1^, and the corresponding results are presented in Figure 3d–f. The electrical output first increases and then decreases with increasing PA film width, reaching the optimum performance at a width of 60 mm. Increasing the PA width enlarges the effective triboelectric contact area and promotes charge generation. When the film width approaches the electrode width (66 mm), the effective electrode area is nearly fully utilized. However, further increasing the PA width to 70 mm causes part of the film to extend beyond the effective electrode region and also increases rotational resistance. Therefore, a PA film width of 60 mm provides the optimum compromise between charge generation and mechanical efficiency.

After optimizing the triboelectric layer, the influence of the rotor configuration is further investigated by varying the number of inner rotors, as shown in Figure 3g–i. The electrical output exhibits an initial increase followed by a gradual decrease as the number of inner rotors increases from 3 to 6, with the optimum performance obtained using five inner rotors. Increasing the number of inner rotors introduces more triboelectric units into each rotation cycle, simultaneously increasing both the effective contact area and the contact frequency. Consequently, more triboelectric charges are generated and transferred during operation, leading to enhanced electrical output. However, when the number of inner rotors further increases to six, the additional rotor mass and friction significantly increase the mechanical resistance of the rotating system, reducing the rotational speed under identical wind conditions. The decrease in rotational efficiency offsets the benefit of the additional triboelectric units, resulting in deteriorated electrical performance. Therefore, five inner rotors achieve the optimum balance between triboelectric enhancement and mechanical resistance.

To further evaluate the consistency of the optimized structure, the electrical outputs of individual TENG units located at different circumferential positions are compared. As shown in Figure 3i, all units exhibit similar output characteristics, although slight variations are observed. In particular, the No. 4 unit delivers the lowest electrical output, whereas the No. 1–3 and No. 5–6 units exhibit relatively higher outputs. These differences are mainly attributed to slight rotor eccentricity, non-uniform wind loading, and local variations in contact pressure, which lead to minor differences in triboelectric contact conditions around the circumference. Nevertheless, the overall output distribution remains relatively uniform, demonstrating the structural reliability of the proposed TENG.

Although the optimized TENG delivers a high output open-circuit voltage, its short-circuit current output remains relatively limited. Therefore, the structural parameters of the EMG are further optimized to improve the overall current output of the hybrid energy harvester. The influence of the magnet number is first investigated while maintaining the wind speed at 6 m s^−1^ and the number of copper coils at six, as illustrated in Figure 3j–l. The electrical output initially increases and subsequently decreases with increasing magnet number, and the optimum performance is achieved using six magnets. Increasing the number of magnets from 4 to 6 increases the number of magnetic poles passing through the coils during each rotation cycle, thereby increasing the magnetic flux variation frequency and enhancing the induced electromotive force [51,52]. However, a further increase to eight magnets strengthens magnetic interactions and increases rotational resistance, resulting in a reduction in rotational speed. Moreover, the mismatch between the numbers of magnets and coils may introduce phase interference among the induced electrical signals, further reducing the output performance. Consequently, six magnets provide the optimum electromagnetic coupling for the present EMG.

The influence of the magnet–coil distance on the electrical performance of the EMG is further investigated, as shown in Figure 3m–o [53,54]. The electrical output gradually decreases as the magnet–coil distance increases from 6 mm to 10 mm. Reducing the separation distance strengthens the magnetic field intensity within the coil region, increases the effective magnetic flux passing through the coils, and suppresses magnetic flux leakage. Consequently, a larger electromotive force is induced in the coils, resulting in enhanced electrical output. Therefore, maintaining a relatively small magnet–coil distance is beneficial for maximizing the electromagnetic generation capability of the EMG.

Conventional rotation-driven TENGs generally employ a single-rotor configuration, in which only one triboelectric component rotates under wind excitation [55,56]. Consequently, the relative sliding velocity between the triboelectric layers is limited by the rotational speed of a single rotor, restricting the charge generation rate and electrical output. To overcome this limitation, an inner–outer counter-rotating architecture is proposed in the present work. Under wind excitation, the upper wind-harvesting assembly drives the inner rotor to rotate clockwise, whereas the lower wind-harvesting assembly simultaneously drives the outer rotor to rotate counterclockwise. The opposite rotational motions substantially increase the relative sliding velocity between the PTFE and PA triboelectric layers without requiring a higher wind speed. As shown in Figure S4, as the wind speed increases from 4 to 10 m s^−1^, the inner rotor speed increases from 48 to 128 rpm, while the outer rotor speed rises from 62 to 159 rpm. Owing to their opposite rotational directions, the corresponding relative rotational speed increases from 110 to 287 rpm, confirming that the counter-rotating configuration effectively enhances the relative motion between the triboelectric interfaces. The enhanced relative motion accelerates triboelectric charge generation and transfer, thereby significantly improving the electrical output of the TENG. Meanwhile, the higher rotational speed also benefits electromagnetic induction in the EMG, enabling the hybrid device to simultaneously achieve high open-circuit voltage and high short-circuit current outputs.

**Figure 4:**
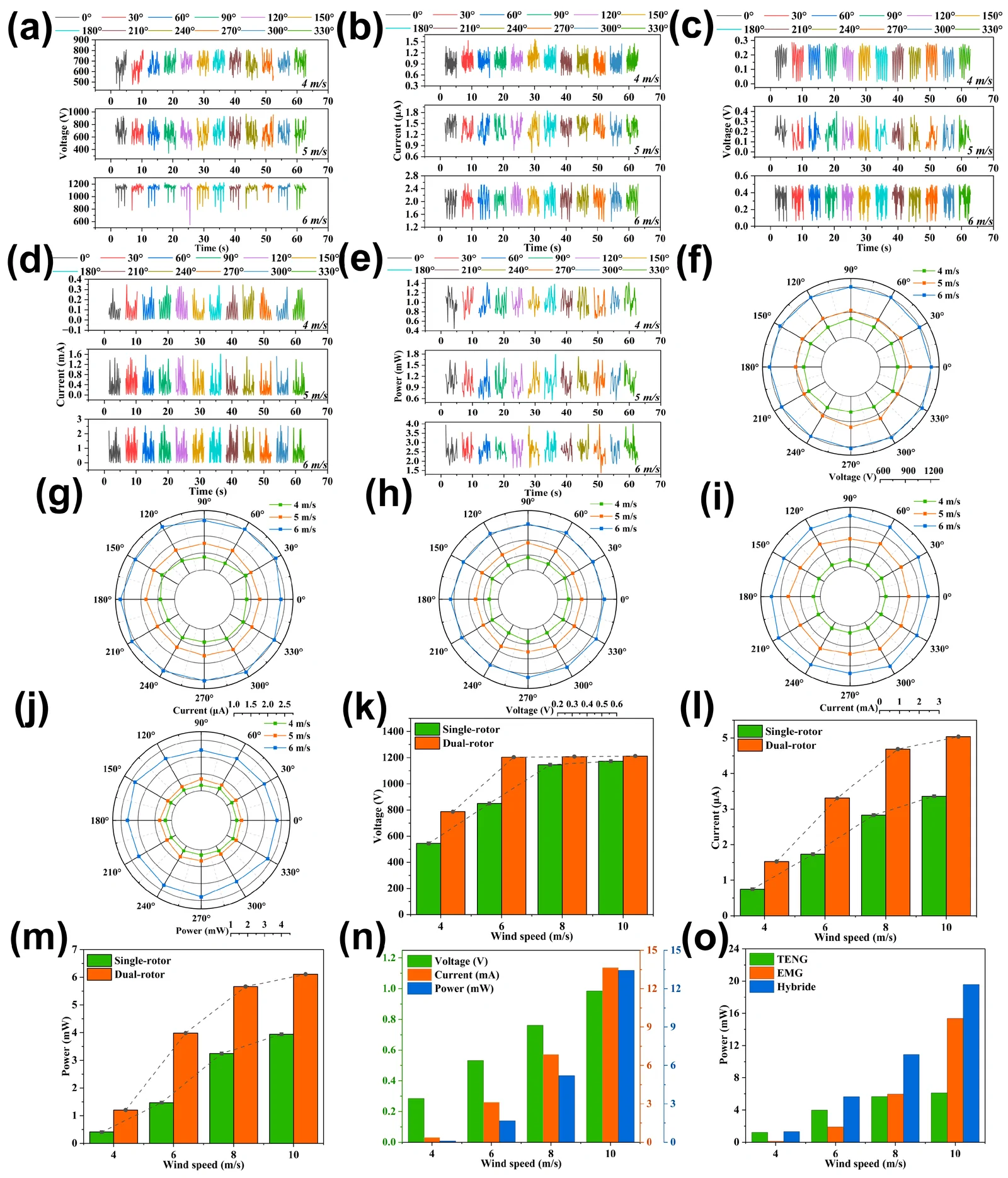
Electrical performance of H-TENG. (**a**) Open-circuit voltage, (**b**) short-circuit current of the TENG on angles ranging from 0 to 360 degrees at wind speeds of 4–6 m s^−1^. (**c**) Open-circuit voltage, (**d**) short-circuit current of the EMG on angles ranging from 0 to 360 degrees at wind speeds of 4–6 m s^−1^. (**e**) Peak output power of H-TENG on angles ranging from 0 to 360 degrees at wind speeds of 4–6 m s^−1^. Directional map of the (**f**) open-circuit voltage, (**g**) short-circuit current of the TENG at wind speeds of 4–6 m s^−1^. Directional map of the (**h**) open-circuit voltage, (**i**) short-circuit current of the EMG at wind speeds of 4–6 m s^−1^. Directional map of the (**j**) peak output power of H-TENG at wind speeds of 4–6 m s^−1^. Comparison of the (**k**) open-circuit voltage, (**l**) short-circuit current, and (**m**) peak output power in TENG between single-rotor system and dual-rotor system at wind speeds of 4–10 m s^−1^. (**n**) Open-circuit voltage, short-circuit current, and output power of the EMG at wind speeds of 4–10 m s^−1^. (**o**) Comparison of output power across the three methods.

To evaluate the omnidirectional wind-energy harvesting capability of the developed H-TENG, its electrical performance is investigated at wind speeds of 4, 5, and 6 m s^−1^ under different wind directions. The wind direction is varied in 30° increments, and the open-circuit voltage, short-circuit current, and output power are measured, as shown in Figure 4a–j. The results show that the device maintains electrical output across the tested wind directions, with limited variation in performance, demonstrating its capability to harvest wind energy omnidirectionally under relatively low wind-speed conditions. The open-circuit voltage of the TENG remains stable at ≈1214.79–1229.23 V, while the EMG remains stable at ≈0.47–0.51 V throughout the range of 0–360°. This interesting property is attributed to the unique dual-rotor structure, resulting in an effective collection of wind energy regardless of its unpredictable direction.

Under identical wind conditions, the dual-rotor configuration consistently delivers higher open-circuit voltage, short-circuit current, and peak output power than the conventional single-rotor design. Especially at 4 m s^−1^, the TENG produces a maximum open-circuit voltage of 786.31 V, a short-circuit current of 1.53 μA, and a peak output power of 1.20 mW, all of which are higher than those obtained in the single-rotor mode (543.92 V, 0.75 μA, and 0.41 mW, respectively). Compared with the single-rotor configuration, the dual-rotor system exhibits an approximately 195.6% increase in peak output power under the same wind conditions. The counter-rotating architecture promotes relative motion between the inner and outer rotors, which may increase the sliding velocity between the triboelectric layers and contribute to enhanced electrical output. Moreover, the experimental results show that the peak output open-circuit voltage is reached at 6 m s^−1^. After reaching a critical wind speed, the open-circuit voltage tends to saturate, which remains stable at ≈1203.94–1212.34 V, demonstrating that the dual-rotor structure improves low-wind-speed adaptability and reduce the air velocity required for the system to operate at its optimum performance.

**Figure 5:**
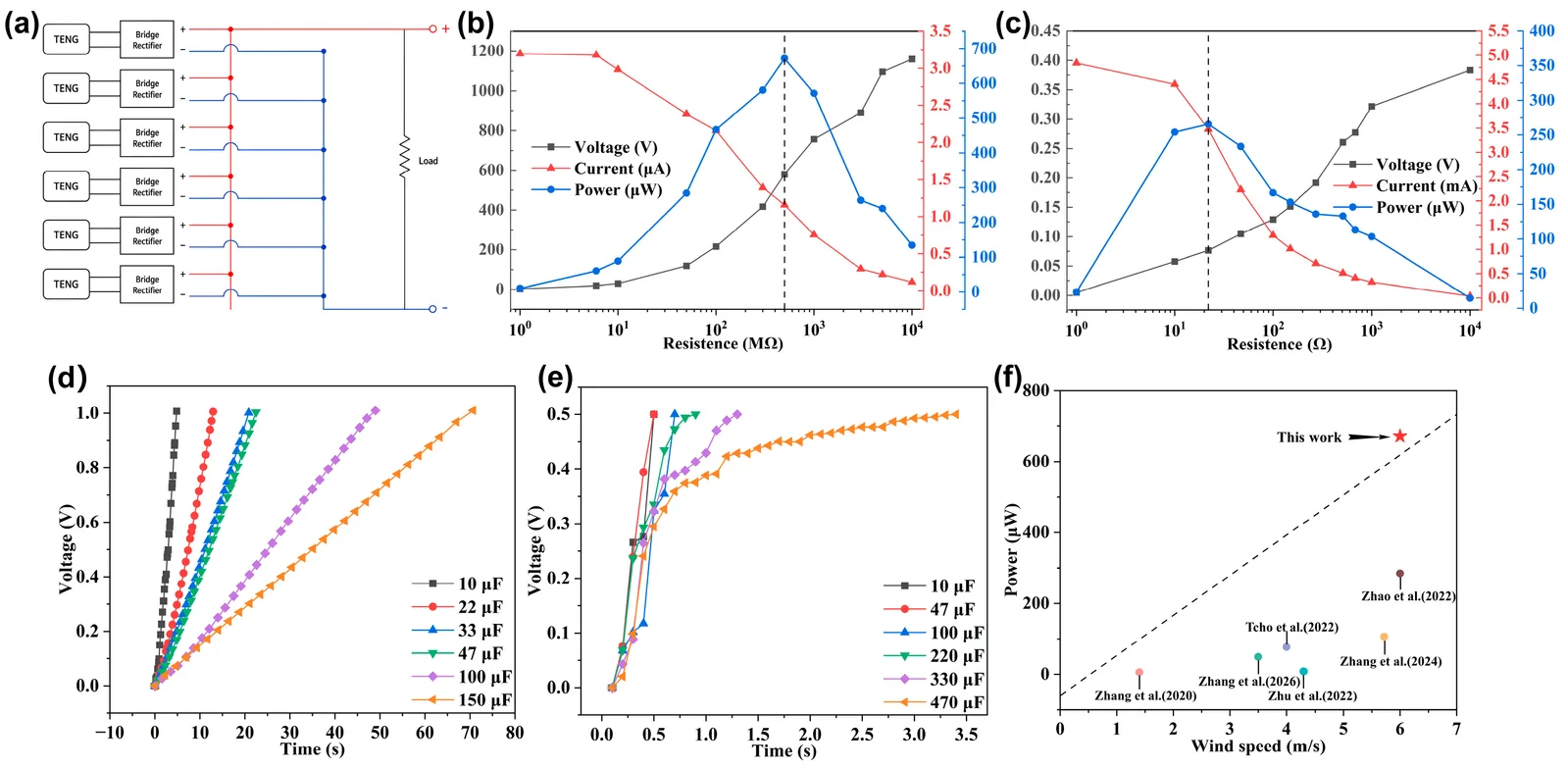
Load-matching and energy storage characteristics of the H-TENG. (**a**) Equivalent circuit of the parallel-connected TENG units. (**b**) Short-circuit current, open-circuit voltage, and peak output power of the TENG as functions of load resistance. (**c**) Short-circuit current, open-circuit voltage, and peak output power of the EMG as functions of load resistance. (**d**) Charging curves of different capacitors charged by the TENG. (**e**) Charging curves of different capacitors charged by the EMG. (**f**) Comparison of our results with previously reported wind energy harvesters, including GTENG [59], VIV-TENG [60], RFW-TENG [61], W-TENG [62], OWTENG [63], and GTEH [64].

To further clarify the advantage of the proposed counter-rotating architecture, the TENG output performance under the two operating modes is directly compared, as shown in Figure 4k–m. Although the optimized TENG provides a high open-circuit voltage output, its short-circuit current output remains relatively limited owing to the intrinsic characteristics of triboelectric nanogenerators. To improve the overall power supply capability of the hybrid system, an EMG is integrated to establish a triboelectric–electromagnetic hybrid energy harvesting mechanism. As shown in Figure 4n, the electrical output of the EMG increases monotonically with increasing wind speed. At a wind speed of 10 m s^−1^, the EMG achieves a maximum open-circuit voltage of 0.99 V, a short-circuit current of 13.64 mA, and a peak output power of 13.44 mW, representing increases of 3.46, 37.56, and 129.8 times, respectively, compared with those measured at 4 m s^−1^. This behavior is attributed to the higher rotational speed of the permanent magnets under stronger wind excitation, which increases the rate of magnetic flux variation through the copper coils and consequently enhances electromagnetic induction according to Faraday’s law. Compared with the TENG, the EMG delivers a substantially higher current output, effectively compensating for the current limitation of the triboelectric generator. As shown in Figure 4o, the synergistic integration of the TENG and EMG enables the hybrid energy harvester to simultaneously achieve high open-circuit voltage and high short-circuit current outputs, thereby significantly improving its practical power supply capability for self-powered electronic systems. A further analysis is performed to clarify the relative contributions of the TENG and EMG to the hybrid electrical output. As shown in Figure S5, the contribution of each unit is evaluated based on its respective output power. The relative contributions are calculated according to Equations (2) and (3):

(1) $$C_{\mathrm{TENG}}=\frac{P_{\mathrm{TENG}}}{P_{\mathrm{TENG}}+P_{\mathrm{EMG}}}\times 100\%$$

(2) $$C_{\mathrm{EMG}}=\frac{P_{\mathrm{EMG}}}{P_{\mathrm{TENG}}+P_{\mathrm{EMG}}}\times 100\%$$

where *C_TENG_* and *C_EMG_* represent the relative contributions of the TENG and EMG, respectively. *P_TENG_* and *P_EMG_* represent the output powers of the TENG and EMG, respectively. At a wind speed of 6 m s^−1^, the TENG and EMG contribute approximately 70.52% and 29.48%, respectively, to the hybrid electrical output. To improve the overall electrical output of the hybrid energy harvester, the six TENG units are connected in parallel, as illustrated in Figure 5a. Previous studies have demonstrated that the connection strategy of multiple TENG units plays a crucial role in determining the overall electrical performance of integrated energy harvesters [57,58]. Compared with series connection, parallel connection effectively increases the output short-circuit current and peak output power while maintaining a relatively stable output open-circuit voltage, making it more suitable for practical energy harvesting applications. To evaluate the output capability of the hybrid energy harvester, the electrical performance is measured under different external load resistances at a constant wind speed of 6 m s^−1^, and the corresponding results are presented in Figure 5b,c. The peak output power is calculated according to Equation (4) [38]:

(3) *P* = *V*^2^/*R*

where P, V, and R represent the peak output power, output open-circuit voltage, and external load resistance, respectively. As the external resistance increases, the output open-circuit voltage gradually increases, whereas the output short-circuit current exhibits the opposite trend, which is consistent with the impedance matching characteristics of TENGs and EMGs. The TENG delivers a peak output power of 671.64 μW at an external load resistance of approximately 500 MΩ, indicating that its internal impedance is close to 500 MΩ. In comparison, the EMG reaches a peak output power of 265.66 μW at an external resistance of approximately 47 Ω, reflecting the fundamentally different electrical characteristics of electromagnetic generators. The large impedance difference further demonstrates the complementary electrical behaviors of the two energy conversion mechanisms, highlighting the advantage of integrating the TENG and EMG into a hybrid energy harvesting system. The energy storage capability of the proposed hybrid harvester is further evaluated using a full-wave bridge rectifier and commercial capacitors with capacitances ranging from 10 to 470 μF, as shown in Figure 5d,e. The charging open-circuit voltage decreases gradually with increasing capacitance because larger capacitors require more stored charge to achieve the same voltage level. Nevertheless, the TENG charges a 10 μF capacitor to more than 1 V within 5 s, while the EMG charges a 100 μF capacitor to above 0.5 V within only 0.7 s. These results demonstrate the rapid energy storage capability of the hybrid device. Specifically, the TENG provides a high charging voltage owing to its high output open-circuit voltage, whereas the EMG significantly accelerates the charging process through its substantially higher current output. The complementary charging characteristics further verify the effectiveness of the triboelectric–electromagnetic hybrid design. To further evaluate the output capability of the proposed device, its electrical performance is compared with those of previously reported wind-driven TENGs, as summarized in Figure 5f. The H-TENG exhibits a noticeably higher output power than most previously reported devices, demonstrating the effectiveness of the synergistic material–structure design strategy based on flexible MWCNTs/PDMS composite electrodes and the counter-rotating architecture for efficient wind energy harvesting.

**Figure 6:**
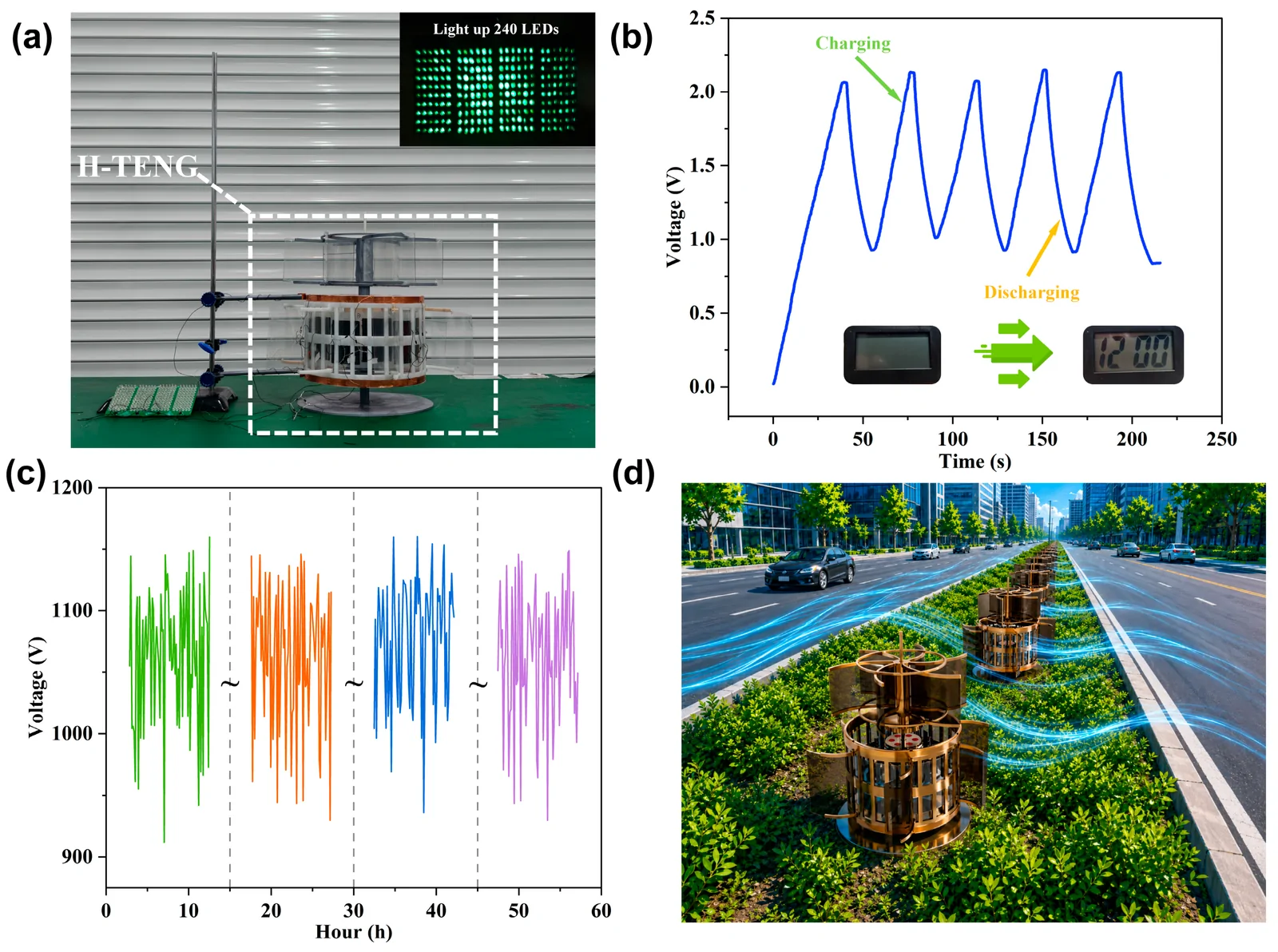
The applications of H-TENG. (**a**) Photograph of LEDs lit directly by the H-TENG. (**b**) Photograph of the digital clock powered by the H-TENG. (**c**) Stability test of the H-TENG. (**d**) Proposed network composed of multiple H-TENG for harvesting large-scale wind energy.

The practical applicability of the hybrid energy harvester is finally demonstrated through several self-powered applications. As shown in Figure 6a and Video S1 (Supporting Information), the generated electrical energy directly powers 240 commercial LEDs under wind excitation produced by electric fans. Furthermore, the hybrid harvester, together with a 33 μF capacitor and bridge rectifiers, continuously powers a commercial digital watch (Figure 6b and Video S2, Supporting Information), demonstrating its capability for supplying power to low-power electronic devices. The operational durability of the H-TENG is further evaluated through a continuous 60 h stability test at a wind speed of 6 m s^−1^, corresponding to approximately 6.048 × 10^5^ relative rotational cycles. As shown in Figure 6c, no noticeable degradation in electrical output is observed throughout the test, demonstrating the device’s operational stability over hundreds of thousands of relative rotations. Owing to its high output performance, good durability, and modular design, the proposed hybrid energy harvester can be readily integrated into distributed energy harvesting networks for large-scale wind energy collection in open environments, as schematically illustrated in Figure 6d.

## 4. Conclusions

In this work, an H-TENG featuring flexible MWCNTs/PDMS composite electrodes and an inner–outer counter-rotating architecture is developed for efficient wind energy harvesting. The synergistic integration of the flexible conductive composite electrode, the counter-rotating dual-rotor configuration, and the hybrid triboelectric–electromagnetic energy conversion mechanism simultaneously enhances the interfacial contact state, relative sliding velocity, and current output, thereby significantly improving the overall energy harvesting performance. Systematic parameter optimization demonstrates that the optimum electrical output is achieved with an MWCNT content of 6 wt%, a PA film thickness of 0.2 mm, a PA film width of 6 cm, and five inner rotors. Under the optimized conditions, the TENG achieves a maximum open-circuit voltage of 1212.34 V, the EMG delivers a maximum output current of 13.64 mA, and the flexible MWCNTs/PDMS electrode and the counter-rotating structure increase the peak output power of the TENG by up to approximately 46.1% and 195.6%, respectively, compared with the conventional copper electrode and single-rotor configuration. The proposed hybrid energy harvester exhibited excellent operational stability during a 60 h continuous test and successfully powered 240 commercial LEDs and a digital watch, demonstrating its practical power supply capability. This work provides a promising material–structure synergistic strategy for high-performance wind energy harvesting and offers a feasible approach for distributed self-powered systems and sustainable ambient energy harvesting.

## Data Availability

The data that support the findings of this study are available from the corresponding author upon reasonable request.

## Supplementary Materials

The following supporting information can be downloaded at: https://www.mdpi.com/article/10.3390/polym18212754/s1. Figure S1. Flowchart for the preparation of MWCNTs/PDMS flexible electrodes, and the insets are the enlarged view of the MWCNTs and MWCNTs/PDMS flexible electrodes. Figure S2. Triboelectric series for some common materials. Figure S3. Resistance and electrical conductivity of MWCNTs/PDMS electrodes with different MWCNTs loadings. Figure S4. Actual rotational speeds of the inner and outer rotors and their relative rotational speed. Figure S5. Percentage contributions of the TENG and EMG to the total hybrid output power. Figure S6. SEM of flexible electrode after electricity generation.

## References

[1] Liu H., Fu H., Sun L., Lee C., Yeatman E.M. (2021). Hybrid Energy Harvesting Technology: From Materials, Structural Design, System Integration to Applications. Renew. Sustain. Energy Rev..

[2] Xu C., Song Y., Han M., Zhang H. (2021). Portable and Wearable Self-Powered Systems Based on Emerging Energy Harvesting Technology. Microsyst. Nanoeng..

[3] Yu R., Feng S., Sun Q., Xu H., Jiang Q., Guo J., Dai B., Cui D., Wang K. (2024). Ambient Energy Harvesters in Wearable Electronics: Fundamentals, Methodologies, and Applications. J. Nanobiotechnol..

[4] Han D.-Y., Song C.K., Lee G., Song W.-J., Park S. (2024). A Comprehensive Review of Battery-Integrated Energy Harvesting Systems. Adv. Mater. Technol..

[5] Perera S.M.H.D., Putrus G., Conlon M., Narayana M., Sunderland K. (2022). Wind Energy Harvesting and Conversion Systems: A Technical Review. Energies.

[6] Omidvarnia F., Sarhadi A. (2024). Nature-Inspired Designs in Wind Energy: A Review. Biomimetics.

[7] McKenna R., Pfenninger S., Heinrichs H., Schmidt J., Staffell I., Bauer C., Gruber K., Hahmann A.N., Jansen M., Klingler M. (2022). High-Resolution Large-Scale Onshore Wind Energy Assessments: A Review of Potential Definitions, Methodologies and Future Research Needs. Renew. Energy.

[8] Msigwa G., Ighalo J.O., Yap P.S. (2022). Considerations on Environmental, Economic, and Energy Impacts of Wind Energy Generation: Projections towards Sustainability Initiatives. Sci. Total Environ..

[9] Li J., Wang G., Yang P., Wen Y., Zhang L., Song R., Hou C. (2024). An Orientation-Adaptive Electromagnetic Energy Harvester Scavenging for Wind-Induced Vibration. Energy.

[10] Lo Monaco M., Russo C., Somà A. (2023). Numerical and Experimental Performance Study of Two-Degrees-of-Freedom Electromagnetic Energy Harvesters. Energy Convers. Manag. X.

[11] Xin M., Jiang X., Xu C., Yang J., Lu C. (2023). Two-Dimensional Omnidirectional Wind Energy Harvester with a Cylindrical Piezoelectric Composite Cantilever. Micromachines.

[12] Wu W., Pan Z., Zhou J., Wang Y., Ma J., Li J., Hu Y., Wen J., Wang X. (2024). Wind-Speed-Adaptive Resonant Piezoelectric Energy Harvester for Offshore Wind Energy Collection. Sensors.

[13] Li J., Chen J., Guo H. (2021). Triboelectric Nanogenerators for Harvesting Wind Energy: Recent Advances and Future Perspectives. Energies.

[14] He L., Zhang C., Zhang B., Yang O., Yuan W., Zhou L., Zhao Z., Wu Z., Wang J., Wang Z.L. (2022). A Dual-Mode Triboelectric Nanogenerator for Wind Energy Harvesting and Self-Powered Wind Speed Monitoring. ACS Nano.

[15] Fan B., Fang J., Jiang S., Li C., Shao J., Liu W. (2024). A Hybrid Energy Harvester Inspired by Bionic Flapping Wing Structure Based on Magnetic Levitation. Rev. Sci. Instrum..

[16] Fang J., Jiang M., Fan B., Chong L., Shao J. (2025). A Hybrid Energy Harvester with Bistable Mechanism Based on Flapping-Wing Structure. Commun. Nonlinear Sci. Numer. Simul..

[17] Perrozzi M.V., Lo Monaco M., Somà A. (2025). Recent Advances in Translational Electromagnetic Energy Harvesting: A Review. Energies.

[18] Ligęza P. (2024). Electromagnetic Energy Harvester Using Pulsating Airflows—Reeds Waving in the Wind. Energies.

[19] Roy S., Kabir M.H., Salauddin M., Halim M.A. (2022). An Electromagnetic Wind Energy Harvester Based on Rotational Magnet Pole-Pairs for Autonomous IoT Applications. Energies.

[20] Zhang Y., Li S., Wang W., Zen P., Li C., Ye Y., He X. (2025). A Review of Electromagnetic Wind Energy Harvesters Based on Flow-Induced Vibrations. Energies.

[21] Li G., Cui J., Liu T., Zheng Y., Hao C., Hao X., Xue C. (2023). Triboelectric-Electromagnetic Hybrid Wind-Energy Harvester with a Low Startup Wind Speed in Urban Self-Powered Sensing. Micromachines.

[22] Muscat A., Bhattacharya S., Zhu Y. (2022). Electromagnetic Vibrational Energy Harvesters: A Review. Sensors.

[23] Du X., Zhang M., Chang H., Wang Y., Yu H. (2022). Micro Windmill Piezoelectric Energy Harvester Based on Vortex-Induced Vibration in Tunnel. Energy.

[24] Hasan M.A.M., Zhu W., Bowen C.R., Wang Z.L., Yang Y. (2024). Triboelectric Nanogenerators for Wind Energy Harvesting. Nat. Rev. Electr. Eng..

[25] Ren Z., Wu L., Pang Y., Zhang W., Yang R., Mao S., Li Y., Zhang H., Cheng T. (2022). Strategies for Effectively Harvesting Wind Energy Based on Triboelectric Nanogenerators. Nano Energy.

[26] Zhao Z., Lu Y., Mi Y., Meng J., Cao X., Wang N. (2022). Structural Flexibility in Triboelectric Nanogenerators: A Review on the Adaptive Design for Self-Powered Systems. Micromachines.

[27] Pragalathan S., Venugopal V. (2025). Brief Review on Nanofiber-Based Triboelectric Material: A Promising Candidate for a Triboelectric Nanogenerator. ACS Omega.

[28] Yang S., He M., Wang Y., Zhang J., Huang X., Yang H. (2024). The Preparation of a Low-Cost, Structurally Simple Triboelectric Nanogenerator and Its Application in Detecting the Movement Characteristics of Gait. Materials.

[29] Hu S., Weber J., Chang S., Xiao G., Lu J., Gao J., Jiang W., Zhang Y., Tao Y. (2022). A Low-Cost Simple Sliding Triboelectric Nanogenerator for Harvesting Energy from Human Activities. Adv. Mater. Technol..

[30] Li X., Yang Q., Ren D., Li Q., Yang H., Zhang X., Xi Y. (2024). A Review of Material Design for High Performance Triboelectric Nanogenerators: Performance Improvement Based on Charge Generation and Charge Loss. Nanoscale Adv..

[31] Kim D.W., Lee J.H., Kim J.K., Jeong U. (2020). Material Aspects of Triboelectric Energy Generation and Sensors. NPG Asia Mater..

[32] Shi B., Wang Q., Su H., Li J., Xie B., Wang P., Qiu J., Wu C., Zhang Y., Zhou X. (2023). Progress in Recent Research on the Design and Use of Triboelectric Nanogenerators for Harvesting Wind Energy. Nano Energy.

[33] Xiang H., Peng L., Yang Q., Wang Z.L., Cao X. (2024). Triboelectric Nanogenerator for High-Entropy Energy, Self-Powered Sensors, and Popular Education. Sci. Adv..

[34] Pace G., Ansaldo A., Serri M., Lauciello S., Bonaccorso F. (2020). Electrode Selection Rules for Enhancing the Performance of Triboelectric Nanogenerators and the Role of Few-Layers Graphene. Nano Energy.

[35] Xie Y., Wang Z., Ren X., Antwi-Afari M.F., Wang Y., Mi H.-Y., Yang B., Liu C., Shen C. (2023). An Internal Electrode Strategy for Enhancing the Stability and Durability of Triboelectric Nanogenerator. Compos. Sci. Technol..

[36] Shin J., Ji S., Cho H., Park J. (2023). Highly Flexible Triboelectric Nanogenerator Using Porous Carbon Nanotube Composites. Polymers.

[37] Zou H., Zhang Y., Guo L., Wang P., He X., Dai G., Zheng H., Chen C., Wang A.C., Xu C. (2019). Quantifying the Triboelectric Series. Nat. Commun..

[38] Huang J., Shao J., Zhong W., Sun C., Zhang G., Chen L., Fang J., Li C., Wang J., Feng X. (2024). A Wind Bell Inspired Triboelectric Nanogenerator for Extremely Low-Speed and Omnidirectional Wind Energy Harvesting. Small Methods.

[39] Wang Y., Chen T., Sun S., Liu X., Hu Z., Lian Z., Liu L., Shi Q., Wang H., Mi J. (2022). A Humidity Resistant and High Performance Triboelectric Nanogenerator Enabled by Vortex-Induced Vibration for Scavenging Wind Energy. Nano Res..

[40] Yu G., Wen J., Li H., Shang Y., Wang Z.L., Chen B. (2024). Vibration-Coupled TENGs from Weak to Ultra-Strong Induced by Vortex for Harvesting Low-Grade Airflow Energy. Nano Energy.

[41] Li Y., Deng H., Wu H., Luo Y., Deng Y., Yuan H., Cui Z., Tang J., Xiong J., Zhang X. (2024). Rotary Wind-Driven Triboelectric Nanogenerator for Self-Powered Airflow Temperature Monitoring of Industrial Equipment. Adv. Sci..

[42] Yong S., Wang J., Yang L., Wang H., Luo H., Liao R., Wang Z.L. (2021). Auto-Switching Self-Powered System for Efficient Broad-Band Wind Energy Harvesting Based on Dual-Rotation Shaft Triboelectric Nanogenerator. Adv. Energy Mater..

[43] Sun C. (2025). Preparation of Kelp-Inspired Triboelectric Nanogenerator Based on PDMS-CNTs Flexible Conductors and Its Application in Wave Energy Harvesting. Master’s Thesis.

[44] Zhou L., Liu X., Zhong W., Pan Q., Sun C., Gu Z., Fang J., Li C., Wang J., Dong X. (2024). Wearable smart silicone belt for human motion monitoring and power generation. Polymers.

[45] Zheng X., Li H., Liu X., Zhong W., Fang J., Li C., Dong X., Shao J. (2026). Wind Bell-Inspired Polymeric Triboelectric Nanogenerator for Efficient Omnidirectional Wind Energy Harvesting at Extremely Low Wind Speeds. Micromachines.

[46] Pace G., Serri M., del Rio Castillo A.E., Ansaldo A., Lauciello S., Prato M., Pasquale L., Luxa J., Mazánek V., Sofer Z. (2021). Nitrogen-Doped Graphene Based Triboelectric Nanogenerators. Nano Energy.

[47] Sidhu J.S., Misra A., Bhardwaj A., Giribabu D. (2026). Investigation of rheological, mechanical, and electrical properties of MWCNT/PDMS composites for extrusion-based direct ink writing. Synth. Met..

[48] Zhang Z., Sun X., Chen Y., Debeli D.K., Guo J. (2018). Comprehensive dependence of triboelectric nanogenerator on dielectric thickness and external impact for high electric outputs. J. Appl. Phys..

[49] Huang Y.-J., Tsai C.-H., Hung C.-W., Chung C.-K. (2024). A PDMS-Al Triboelectric Nanogenerator Using Two-Pulse Laser to Enhance Effective Contact Area and Its Application. Polymers.

[50] Sun C., Liu X., Zhong W., Pan Q., Chen L., Zhang G., Wang J., Dong X., Shao J. (2025). A Kelp Inspired High-Power Density Triboelectric Nanogenerator with Stacking Structure for Multiple Directional Ocean Wave Energy Harvesting. Adv. Mater. Technol..

[51] Li Y., Li J., Liu Z., Hu H., Hu J., Wang J., He Z. (2023). Theoretical and Experimental Investigation of Magnet and Coil Arrays Optimization for Power Density Improvement in Electromagnetic Vibration Energy Harvesters. Energy Convers. Manag..

[52] Li Z., Liu Y., Yin P., Peng Y., Luo J., Xie S., Pu H. (2021). Constituting Abrupt Magnetic Flux Density Change for Power Density Improvement in Electromagnetic Energy Harvesting. Int. J. Mech. Sci..

[53] Peng Y., Zhang D., Luo J., Xie S., Pu H., Li Z. (2021). Power Density Improvement Based on Investigation of Initial Relative Position in an Electromagnetic Energy Harvester with Self-Powered Applications. Smart Mater. Struct..

[54] Mou K., Ji X., Li X., Zhou H., Wu Y., Fang Y. (2025). A Green Electromagnetic Energy Harvester with Up-Frequency and Unidirectional Rotation for Smart Pavement. Materials.

[55] Liao W., Su X., Li B., Li H., Zhang K., Fang F., Huang X. (2025). Mimosa-Inspired Tribo-Electromagnetic Nanogenerator for Adaptive Wind Energy Harvesting and Intelligent Wind Monitoring. Nano Energy.

[56] Lee D., Cho S., Kim Y.-S., Jang Y., Oh D., Ra Y., Jang S., Hwang S., Chae J., Choi K. (2025). A Self-Adaptive Triboelectric–Electromagnetic Hybrid Wind Energy Harvester for Sustainable Air Quality Control and Environmental Monitoring. Nano Energy.

[57] He X., Zhang H., Jiang J., Liu X. (2023). Output Characteristics of Series-Parallel Triboelectric Nanogenerators. Nanotechnology.

[58] Li Z., Gan W.C., Tang L., Aw K.C. (2023). Fundamental Understanding of Multicellular Triboelectric Nanogenerator with Different Electrical Configurations. Micromachines.

[59] Zhang L., Meng B., Xia Y., Deng Z., Dai H., Hagedorn P., Peng Z., Wang L. (2020). Galloping Triboelectric Nanogenerator for Energy Harvesting under Low Wind Speed. Nano Energy.

[60] Zhang C., Zhang T., Chen G., Li C., Li X. (2026). Vortex-Driven Triboelectric Nanogenerator for Multidirectional Wind Energy Collection with Humidity Resistance. iEnergy.

[61] Tcho I.W., Kim W.G., Kim J.K., Kim D.W., Yun S.Y., Son J.H., Choi Y.K. (2022). A Flutter-Driven Triboelectric Nanogenerator for Harvesting Energy of Gentle Breezes with a Rear-Fixed Fluttering Film. Nano Energy.

[62] Zhu W., Hu C., Bowen C.R., Wang Z.L., Yang Y. (2022). Scavenging Low-Speed Breeze Wind Energy Using a Triboelectric Nanogenerator Installed inside a Square Variable Diameter Channel. Nano Energy.

[63] Zhang L., He Y., Meng B., Dai H., Abdelkefi A., Wang L. (2024). Unlocking Multidirectional and Broadband Wind Energy Harvesting with Triboelectric Nanogenerator and Vortex-Induced Vibration of Sphere. Appl. Math. Mech. Engl. Ed..

[64] Zhao C., Hu G., Yang Y. (2022). A Cantilever-Type Vibro-Impact Triboelectric Energy Harvester for Wind Energy Harvesting. Mech. Syst. Signal Process..

