# Supplementary material for: The Flexible MWCNTs-PDMS Electrode-Based Counter-Rotating Hybrid Triboelectric–Electromagnetic Nanogenerator for Effective Wind Energy Harvesting
Source: Polymers (Basel). 2026 Nov 5;18(21):2754. doi: 10.3390/polym18212754 (PMC13765730; doi:10.3390/polym18212754)
Supplement: Supplementary file 1 [file polymers-18-02754-s001.zip › polymers-4494974-supplementary.pdf]

Supporting information

The Flexible MWCNTs-PDMS Electrode-Based Counter-Rotating Hybrid Triboelectric–Electromagnetic Nanogenerator for Effective Wind Energy Harvesting

Yao Zhu [1], Xue Liu [2], Wei Zhong [1,*], Jiwen Fang [1], Chong Li [1], Xiaohong Dong [3], and Jiang Shao [1,*]

This file includes:

Figure S1. Flowchart for the preparation of MWCNTs/PDMS flexible electrodes, and the insets are the enlarged view of the MWCNTs and MWCNTs/PDMS flexible electrodes.

Figure S2. Triboelectric series for some common materials.

Figure S3. Resistance and electrical conductivity of MWCNTs/PDMS electrodes with different MWCNTs loadings.

Figure S4. Actual rotational speeds of the inner and outer rotors and their relative rotational speed.

Figure S5. Percentage contributions of the TENG and EMG to the total hybrid output power.

Figure S6. SEM of flexible electrode after electricity generation.

---

1 The College of Mechanical Engineering, Jiangsu University of Science and Technology, Zhenjiang 212000, China

2 The College of Chemistry and Molecular Sciences, Henan University, Kaifeng 475001, China

3 Yangzhou Chenhua New Materials Co., Ltd., Yangzhou 225000, China

* Correspondence: zhongwei@just.edu.cn (W.Z.); jiangshao@just.edu.cn (J.S.)

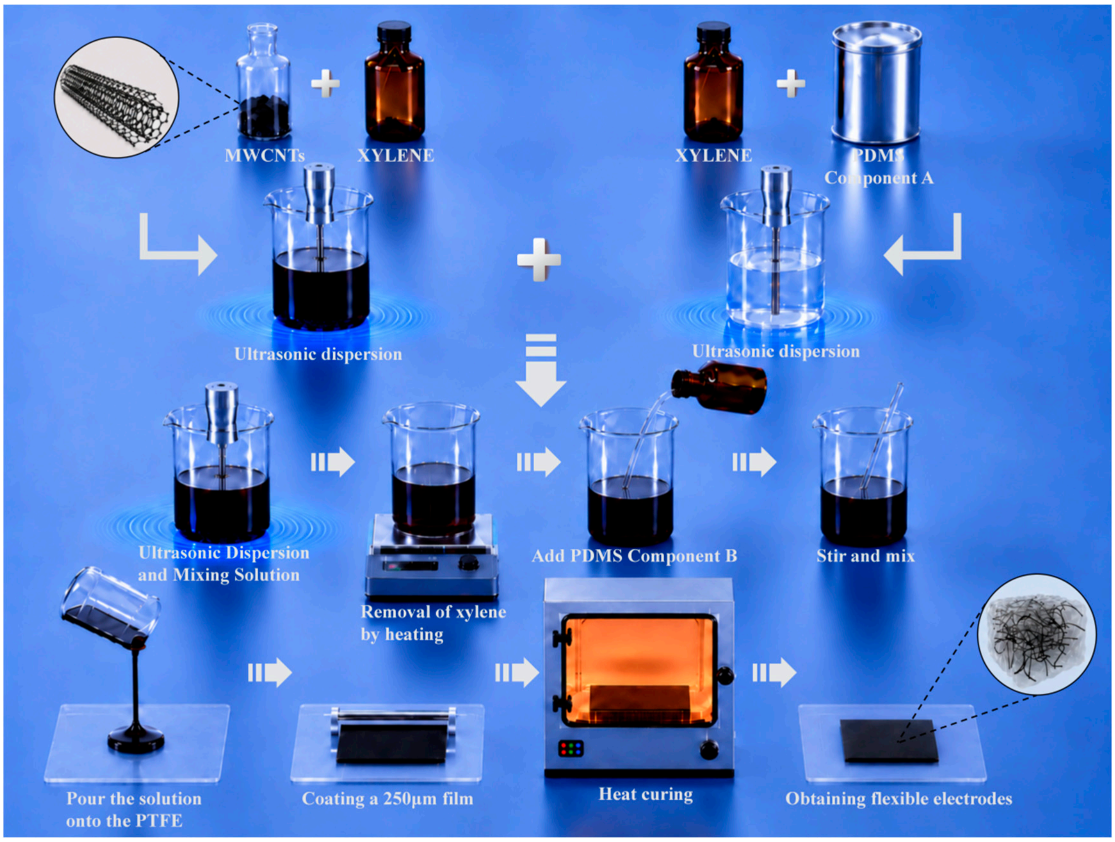

Figure S1. Flowchart for the preparation of MWCNTs/PDMS flexible electrodes, and the insets are the enlarged view of the MWCNTs and MWCNTs/PDMS flexible electrodes.

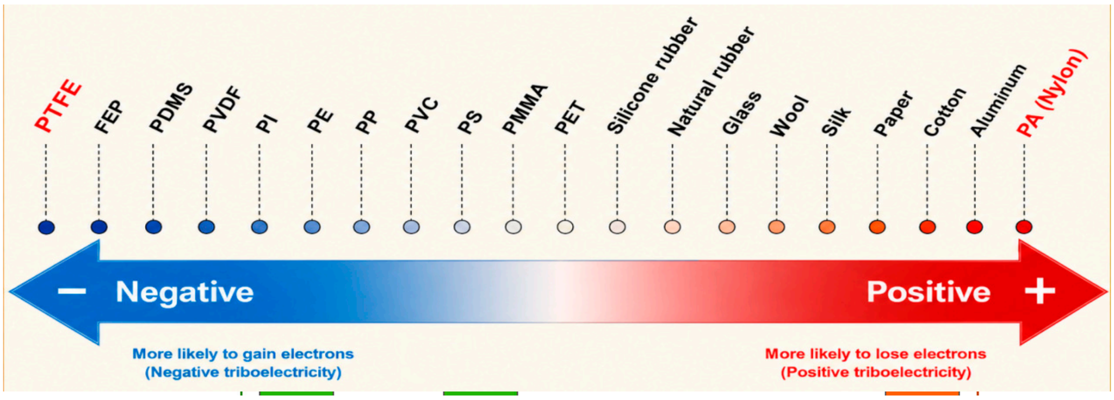

Figure S2. Triboelectric series for some common materials.

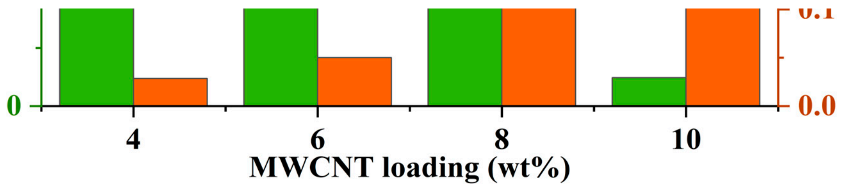

Figure S3. Resistance and electrical conductivity of MWCNTs/PDMS electrodes with different MWCNT loadings.

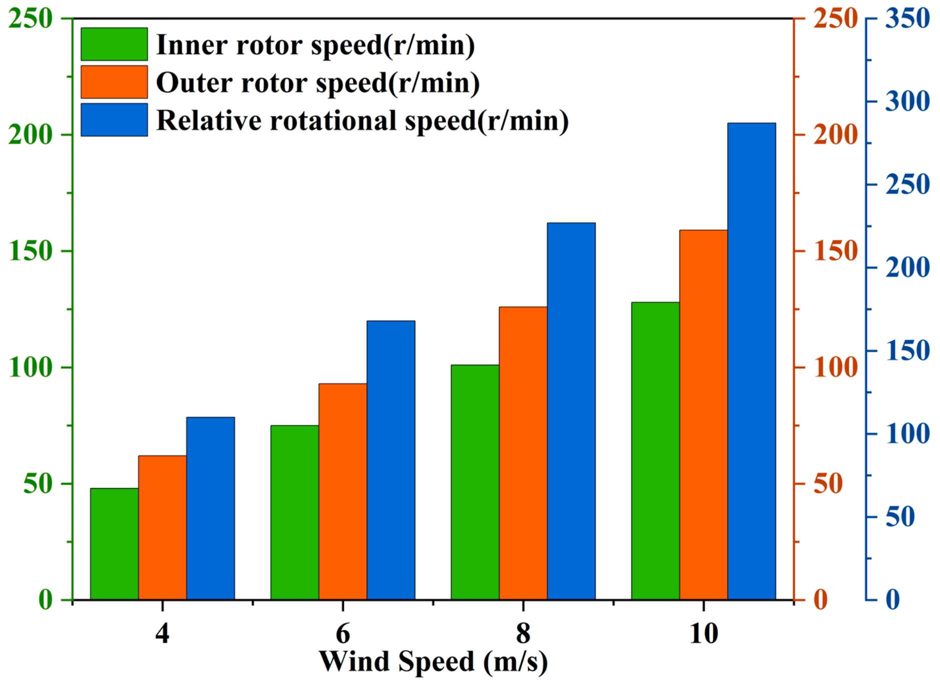

Figure S4. Actual rotational speeds of the inner and outer rotors and their relative rotational speed.

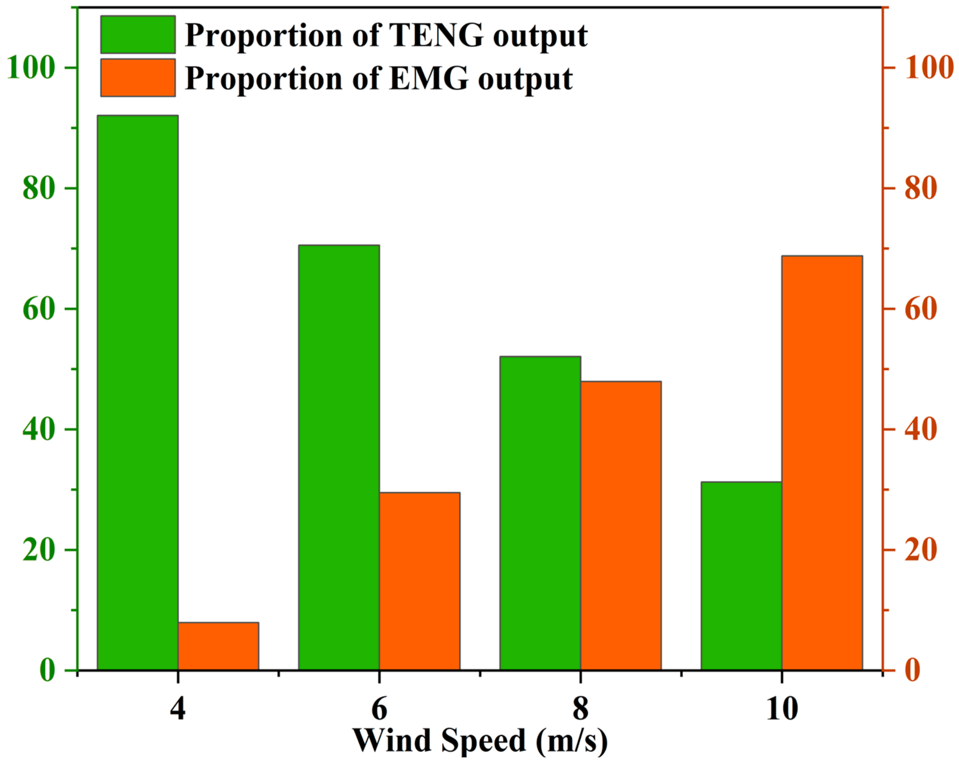

Figure S5. Percentage contributions of the TENG and EMG to the total hybrid output power.

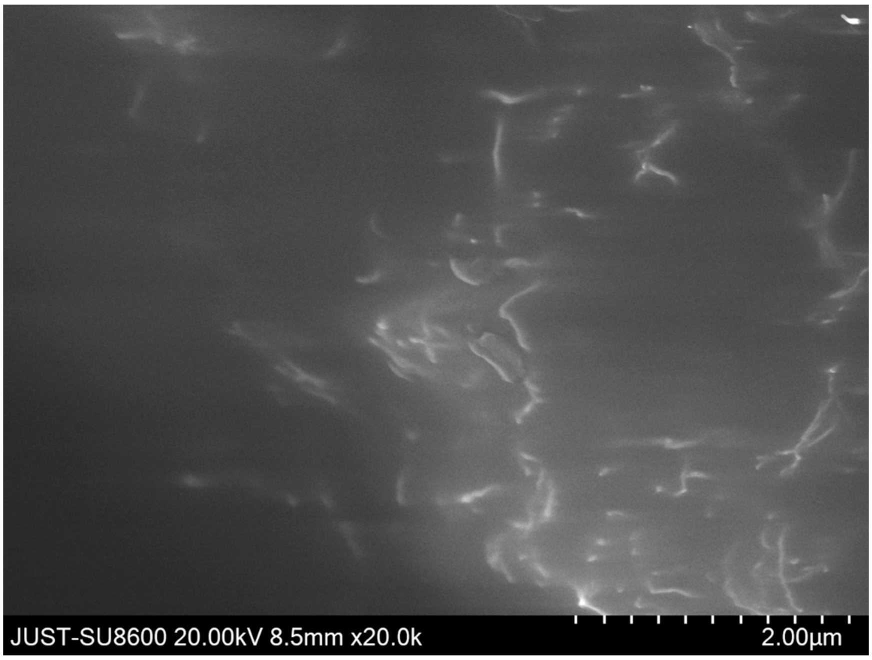

Figure S6. SEM of flexible electrode after electricity generation.
